# Influence of occupational stress on job satisfaction among university workers

**DOI:** 10.1192/j.eurpsy.2025.1631

**Published:** 2025-08-26

**Authors:** N. Rmadi, A. Hrairi, I. Makni, N. Kotti, A. Kchaou, M. Hajjaji, K. Jmal Hammami

**Affiliations:** 1 Occupational medicine department, Hedi chaker university hospital, University of Sfax; 2 Family medicine department, University of Sfax, Sfax, Tunisia

## Abstract

**Introduction:**

Occupational stress has been identified as a significant predictor of job satisfaction among university staff.

**Objectives:**

This paper aims to find out the relationship between occupational stress and job satisfaction among university workers in Sfax, Tunisia.

**Methods:**

We conducted a cross-sectional study, during the period from September 2022 to October 2023. We used a self-administered questionnaire distributed to university staff. The questionnaire included socio-professional characteristics and assessment of occupational stress using the Work Stress Screener (WoSS-13). Job satisfaction was rated on a 5-point Likert scale from 1 (extremely dissatisfied) to 5 (extremely satisfied).

**Results:**

The average age of the workers was 49.31±8. years. There is a slight female predominance (78 (53.1%)). Among the respondents, 57 (38.3%) were very to extremely satisfied. The medians of WoSS-13 subscales were 11 for WOSS-A1, 6 for WOSS-A2 and 3 for WOSS-B. Job satisfaction was positively associated with WOSS-A1 and WOSS-A2 (positive stress) but negatively associated with WOSS-B (negative stress). In bivariate analysis, being extremely satisfied was positively associated to WOSS-A1 and WOSS-A2 (p=0.000 for each respectively). However, it was negatively associated to WOSS-B (p=0.000).

**Conclusions:**

Occupational stress has a significant impact on job satisfaction among university workers. Addressing job stress through targeted interventions can improve job satisfaction, productivity, and overall well-being among university staff.

**Disclosure of Interest:**

None Declared

